# Assessing Children's Anxiety Using the Modified Short State-Trait Anxiety Inventory and Talking Mats: A Pilot Study

**DOI:** 10.1155/2012/932570

**Published:** 2012-09-09

**Authors:** Stefan Nilsson, Margret Buchholz, Gunilla Thunberg

**Affiliations:** ^1^School of Health Sciences, Borås University, 501 90 Borås, Sweden; ^2^DART—Centre for AAC and Assistive Technology, The Queen Silvia Children's Hospital, Sahlgrenska University Hospital, 41345 Gothenburg, Sweden

## Abstract

*Background*. Preoperative anxiety complicates treatment and requires assessment by nurses in children. Children, with or without disability, are helped when pictures are used to support communication. The purpose of this pilot study was to test the reliability and validity of the modified short State-Trait Anxiety Inventory (STAI) using a modified Talking Mats method in children undergoing day surgeries. *Method*. A modified short STAI with pictorial support along the lines of the Talking Mats method was pre- and postoperatively administered to 42 typically developing children aged three to nine years. The parents assessed the children's anxiety, simultaneously and independently, by scoring the short STAI by proxy. *Results*. The modified short STAI showed moderate internal consistency and good construct validity in the age group seven to nine years. *Conclusions*. The results of this study support the use of the instrument for self-reports in children aged seven to nine years. Future research will explore the possibilities of also using this instrument for children with cognitive and communicative difficulties.

## 1. Introduction

It is a main concern to reduce preoperative anxiety in children, because it is associated with painful effects on postoperative recovery [1, 2]. Preoperative anxiety is also correlated with adverse postoperative behavioral abnormalities [3]. It is important for nurses to measure children's emotional responses to surgery when planning appropriate nursing interventions [4]. It is also important for nurses to assess children's anxiety with a validated instrument. Children's emotions and anxiety behavior can sometimes be measured on an observational scale [5]. However, as children grow up, their behavioral expressions will be partially under voluntary control, and self-reports and behavioral measurements are only modestly correlated in research. Behavioral assessments, as well as self-reports, have several validity problems [6]. It is a challenge to use self-reports to assess children's anxiety. Children's levels of distress and their cognitive and communicative competence are essential concerns when nurses need valid self-reports [7]. It is also a challenge to obtain the child's perspective and use a self-report if the child has a cognitive or communicative disability [8]. Despite the methodological challenges of administering self-reports, it is still considered the golden standard [9]. Parents' and nurses' perceptions of children's emotions should only be considered an estimate of the emotions experienced by children, as they are not the children's own self-reports [10]. For this reason it is important to develop and test instruments that could be used by children themselves. Children's opportunities to participate in decisions about their care increase when they are able to communicate their emotions, and this also enables nurses to have a child's perspective. Children should be supported to maintain their competence and be able to communicate their thoughts when they undergo examinations and treatments [11].

Several self-reports have been tested in children, such as the Faces Pain Scale (FPS) for pain intensity that has been validated from four years [12]. However, this needs to be reevaluated, as other research has questioned the validity of the FPS in four-five-year-old children [13, 14]. Other instruments that measure anxiety and fear have also been validated in children above four-to-five, for example, the Children's Fear Scale [15] and the Facial Affective Scale [16, 17]. A visual analogue anxiety scale has also been validated to measure anxiety in children [18], adolescents [19], and adults [20]. All of these instruments assess a single item, which is vulnerable if the child does not specifically understand the item. It is valuable to find instruments with more variables that identify children's feelings.

The State-Trait Anxiety Inventory for Children State form (STAIC-S) was developed by Spielberger and contains 20 items, and it is one of the most frequently used self-report instruments for evaluating children's anxiety. The STAIC-S offers high reliability and satisfactory validity [21]. The STAIC-S is widely used to assess children's anxiety. For example, it has previously been used in conjunction with surgery [22]. Despite the wide use of STAIC-S, it has several limitations, such as its length and complexity of use. Children with limited linguistic competency and/or reading ability also need help from their parents to fill out the STAIC-S, risking its reliability and validity. For example, in a study, 10 out of 16 children (62.5%) needed help from their parents to fill out the form [23]. Another study also showed that children neither completed nor recognized all of the items [24]. A short form of the STAI was used and validated in children aged 5–16 years [23] (Table 1). The short form of the STAI includes six statements. The range for the short STAI is 6 to 24 points, with 6 points signifying no anxiety and 24 points signifying the highest level of anxiety [25]. The short STAI has mainly been used in adults, though a few studies have also confirmed its usability in school-aged children [26, 27]. The construct validity was confirmed, since the scores on the short STAI decreased significantly after a medical procedure that was associated with anxiety. The concurrent validity was established, and the short STAI was compared with the STAIC-S. The Spearman correlation coefficient showed 0.88 before and 0.75 after a medical procedure. The Cronbach's alpha coefficient of the short STAI was 0.82 in this study. One limitation of the results was that 7 out of 16 children (44%) needed help from their parents to fill out the instrument [23].

This points to a need to develop an anxiety scale that places fewer demands on reading ability, language, and cognition to include children with cognitive and/or communicative problems. Most children develop the ability to understand self-reports between the ages of three and seven years [28]. Age as a marker of the usefulness of a self-report instrument is a statistically significant predictor: preschool-aged children are often less likely to understand a self-report than a seven-year old [14]. Children also usually prefer to score their emotions on face scales compared with other methods [29]. In order to better adapt to the cognitive and communicative abilities and styles of younger children and to increase the options for children with disabilities to express their anxiety independently, the Talking Mats method vas applied to the short STAI. Talking Mats form a pictorial framework based on three sets of picture symbols (topic, scale, options) that are presented to individuals with cognitive and/or communicative difficulties [30]. The method has been shown, in earlier studies, to allow people with cognitive and communicative difficulties to express their thoughts and views [31–35]. So far, the research on the Talking Mats method has mainly been conducted with adults with different kinds of disabilities.

The aim of this pilot study was to test the reliability and validity of the modified short STAI using the pictorial framework Talking Mats in typically developing children undergoing day surgeries. An important aspect of the validation was to investigate the scale's applicability with respect to age. The pilot study is part of the larger project KomHIT—Communication in child hospital care settings using augmentative strategies and IT—that has the purpose to investigate and improve the communication situation of children with communicative disabilities during hospital care. KomHIT conforms to the idea of inclusion and tries to create a communicative hospital environment in which the methods and tools developed are accessible and applied more generally to all children. The use of the modified STAI and Talking Mats to also enable children with communicative disabilities to express anxiety will be investigated and reported in a future study.

## 2. Method

This pilot study had a quantitative approach and tested concurrent validity, construct validity, and internal consistency.

During a study period of four months children, aged three to nine years, and their parents were consecutively recruited from a day surgery unit at the Queen Silvia Children's Hospital, Gothenburg, Sweden. The data collection went on for 21 predetermined days. The surgical interventions were not preselected in this study. Based on the literature of von Baeyer et al. [14], which studies the relationship between cognitive development and self-reporting, the children were divided into three age groups, that is, three to four years, five to six years, and seven to nine years. Children with cognitive and communicative impairments were excluded from the study, as were children and parents who did not have a good command of Swedish. As mentioned, these will be studied in the next phase of this project.

**Figure 1:**
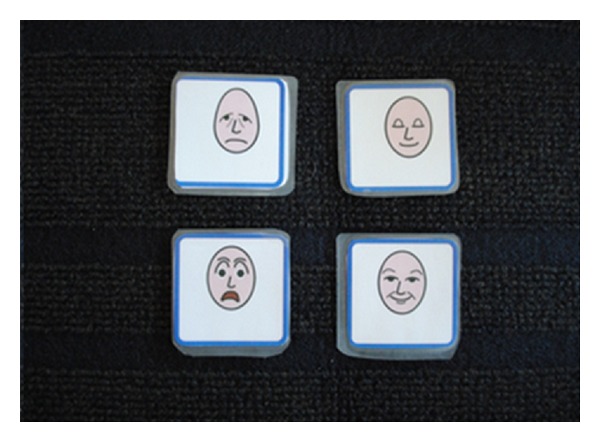
Four faces using Widgit Rebus symbols.

**Figure 2:**
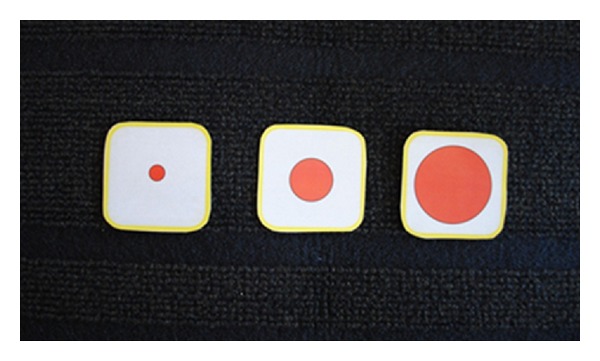
Three circles of different sizes (small, medium, and large).

**Figure 3:**
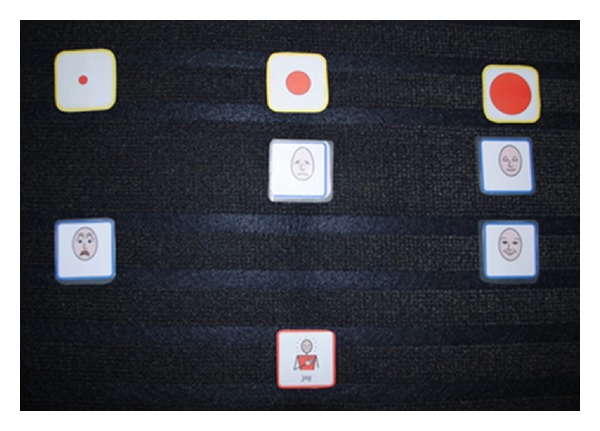
The modified short State-Trait Anxiety Inventory (STAI) using a modified Talking Mats method.

In a prestudy, five children were told to report the usability of six pictures based on short STAI. In these cases, six faces were more than necessary. Based on these reflections, a team of experts of Augmentative and Alternative Communication (AAC) and pediatric nursing were told to select four pictures that matched the six items in short STAI. A modified short STAI was developed in which six statements were transformed into four faces using Widgit Rebus symbols [36]. These pictures were chosen by the researchers to fit the feelings in the original short STAI (Figure 1). Two of the faces demonstrate negative feelings, that is, tenseness and fear. The other two faces demonstrate positive feelings, that is, calmness and happiness. The child places each of these four faces on a mat using a modified Talking Mats method [30]. The Talking Mats method aims to reflect individual preferences and concerns. Three circles of different size (small, medium, and large) were used to signify “not at all,” “moderately,” and “very much” (Figure 2). The child is given the facial expression cards one at a time and is then instructed to place each one according to his or her preference (Figure 3). Finally, the instrument gives a scoring on the child's level of anxiety. The range for this instrument is 4 to 12 points, with 4 points signifying no anxiety and 12 points signifying the highest level of anxiety.

All of the children who underwent investigations and treatments during the study period scored their anxiety before and after the surgery on the modified short STAI using the modified Talking Mats method. One researcher showed each of the four facial expressions to the children in a predetermined order. The children valued each expression according to their own feelings and placed the card underneath one of three different-sized circles (small, medium, and large). Finally, the researcher counted each facial expression and calculated the total, which ranged from 4 to 12. One parent of each child assessed the child's anxiety, simultaneously and independently, by scoring the short STAI by proxy.

Nonparametric tests were used, as the data were not normally distributed and the measurements lacked firm interval quality [37]. The Spearman's correlation analysis was used to compare the short STAI by proxy scores with the children's self-reports of anxiety. A correlation of 0.5 or above was considered sufficient to demonstrate the concurrent validity [38]. The Wilcoxon signed rank test was used to compare changes in the modified short STAI scores before and after the surgery. The use of modified short STAI scores before and after day surgery confirmed the construct validity in this study since another study has evaluated that short STAI scores decreased after day surgery in children aged 7–16 years [26]. A *P* value below 0.05 was considered significant to demonstrate the construct validity. The agreement with internal consistency was analyzed by both Cronbach's alpha and Pearson's correlation, for a correlation between each item and the scale total, omitting that item. A Cronbach's alpha coefficient of 0.7 or above shows high internal consistency, and any item with a Pearson's correlation of less than 0.20 needs to be eliminated or rewritten [39]. In an earlier pilot study, a significant decrease in anxiety, that is, *P* < 0.05 (Wilcoxon signed rank test), was found when 14 children, aged 5 to 16 years, scored their anxiety on the short STAI before and after a surgical or medical procedure [23]. It was decided in this pilot study that each study group should consist of 14 participants who scored their anxiety on the modified short STAI before and after day surgery. 

### 2.1. Ethical Considerations

The study was approved by the Regional Medical Ethics Review Board of Gothenburg. The parents were provided with identical oral and written information about the study, and the children were also given appropriate information with respect to age. Oral informed consent was obtained from all the participants, and written consent was collected from all the parents. The parents and children who participated were thoroughly informed about the purpose of the study and about their right to withdraw from participation at any time. This study focused on frightened children and parents, and it was important to inform them explicitly about the voluntariness to participate in this study. However, this study did not change any circumstances for these children and parents. On the contrary, the researcher in this study asked the children to express their feelings. An earlier study demonstrated that this was beneficial to children in most circumstances [40]. All identities have been kept confidential.

## 3. Results

Forty-two children, aged three to nine, participated in this study. This number of participants fulfilled the criterion of power in this pilot study. The first children and parents in the day surgery who fulfilled the inclusion criteria participated, and all the children and parents agreed to participate in this study. No child or parent declined to participate due to distress, illness, or emotional state. The demographic data are shown in Table 2.

### 3.1. Concurrent Validity

The distribution of the children's scoring before and after the procedure is presented in Table 3. A significant correlation was found between the short STAI by proxy scores and the children's self-reports on the modified short STAI using Talking Mats in the age group three to four years (*r* = 0.50). No other study group showed a significant correlation between the short STAI by proxy and the modified short STAI using Talking Mats.

### 3.2. Construct Validity

The median-modified short STAI scores decreased significantly (*P* 0.004) from 6.5 before the surgery to 5 after the surgery in the age group seven to nine years. The parents' median short STAI by proxy scores also decreased significantly (*P* 0.037) from 13 before the surgery to 9.5 after the surgery. No other scores decreased significantly for the children in the other age groups. 

### 3.3. Internal Consistency

The Cronbach's alpha coefficient was 0.25 for the modified short STAI scores in the age group three to four years, 0.59 in the age group five to six years, and 0.68 in the age group seven to nine years. The Pearson's correlation coefficient showed acceptable correlation between items in the age groups seven to nine years (*r* = 0.36–0.56) and five to six years (*r* = 0.26–0.55) but lower and not suitable items in the age group three to four years (*r* = −0.01–0.30).

## 4. Discussion

The results of this study initially verified the internal consistency, that is, a Pearson's correlation coefficient above 0.20, of the modified short STAI using Talking Mats in the age group seven to nine years. The internal consistency was not very good for the age group five to six years and even lower in the age group three to four years. Hitherto, the statistics partially support internal consistency and confirm construct validity for the modified short STAI scores in children above seven years. 

This study did not confirm whether this modified instrument is usable in children below seven years. This result is consistent with previous studies, which have evaluated facial expressions that symbolize emotions. One study, for example, confirmed that school-aged children could manage self-reports for the purpose of assessing anxiety better than younger children [14]. It may therefore be necessary to exclude younger children from using self-report scales [14]. 

This study indicated that the Talking Mats method might contribute to alleviating anxiety in children above seven years who undergo surgery. It is valuable that children are able to express their emotions, because this helps the health professionals to find strategies that may reduce children's level of anxiety. Music medicine is an example of a strategy that nurses could offer to reduce anxiety when children undergo procedures [41] or day surgery [26].

The use of the Talking Mats method also helps children to show their emotions without involvement from the nurse. Children select each picture themselves and are able to choose independently the level of both positive and negative emotions. This is a promising method to use in children, but hitherto no report exists in the literature about the use of Talking Mats in children [42]. 

The advantage of our instrument, except for the use of pictorial support, is that it measures more than one dimension of anxiety, that is, four feelings. Four facial expressions help the researcher to validate the child's understanding of the face expressions. This is probably also the only face scale that does not only measure negative feelings, such as the Children's Fear Scale [15], the Facial Affective Scale [16, 17], and a visual analogue anxiety scale [18]. This instrument actually lets the child measure positive feelings, which probably helps him or her to use positive coping strategies. It is known that negative expressions from others, such as parents, can negatively influence children's experiences [43]. Future research will explore the possibilities of also using this instrument for children with cognitive and communicative difficulties.

One limitation was the study sample. It would therefore be worthwhile to repeat this study with a larger sample. It is also known that self-reports by proxy risk lack validity and reliability [10], and this study failed to show concurrent validity with the short STAI by proxy scores. It would be of value to change the study design of future research and compare children's scores on the modified short STAI using Talking Mats with another self-report scale. 

The range of surgical interventions differed in the data collection, which means that some might have been experienced as more traumatic than others. However, the aim was to test the instrument in different circumstances and levels of anxiety. It is important to assess validity and reliability of instruments at both low and high levels of anxiety.

This initial validation study can result in recommendations to nurses in their care of children who undergo medical and surgical procedures. The results demonstrate that the modified short STAI using Talking Mats can probably be used to measure anxiety in children above seven years. However, this new instrument needs to be investigated further to confirm its validity and reliability. The next step is to also validate this instrument for children with cognitive and communicative difficulties.

## 5. Conclusion

This study showed that children above seven years were able to use the modified short STAI with Talking Mats when they underwent day surgery. It is important to encourage the use of self-reports because parents by proxy scores were not valid to use in this context.

## Figures and Tables

**Table 1:** Short STAI (state-trait anxiety inventory).

	Not at all	Somewhat	Moderately	Very much
(1) I feel calm	1	2	3	4
(2) I am tense	1	2	3	4
(3) I feel upset	1	2	3	4
(4) I am relaxed	1	2	3	4
(5) I feel content	1	2	3	4
(6) I am worried	1	2	3	4

**Table 2:** 

	3-4 y	5-6 y	7–9 y
	*n* = 14	*n* = 14	*n* = 14
Parents			
Mothers	11	9	9
Fathers	3	5	5
Children			
Girls	7	6	6
Boys	7	8	8
Age (Median)	4	6	8
Surgery			
Injection	2	3	2
Ear	3	2	0
Colo-/Gastroscopy	3	1	5
Hernia inguinalis	4	4	0
Retentio testis	1	1	2
Other	1	3	5

**Table 3:** 

	Short STAI by proxy	Modified short STAI	Spearman's correlation	Wilcoxon sign rank test
	Median (range)	Median (range)	(*r*)	(*P*-value)
3-4 years				
Parents before	10.5 (6–17)			
after	10 (6–23)			0.67
Children before		4.5 (4–8)		
after		5 (4–8)		0.17
Parents-Children before			−0.28	
Parents-Children after			0.50^▲^	
5-6 years				
Parents before	10 (6–18)			
after	8.5 (6–19)			0.21
Children before		5 (4–7)		
after		4.5 (4–9)		0.67
Parents-Children before			0.19	
Parents-Children after			0.27	
7–9 years				
Parents before	13 (6–19)			
after	9.5 (6–16)			0.037*
Children before		6.5 (5–11)		
after		5 (4–6)		0.004**
Parents-Children before			0.47	
Parents-Children after			0.15	

*P < 0.05, **P < 0.01,^▲^ *r* = ≥ 0.5.

## References

[1] Adams HA (2011). A perioperative education program for pediatric patients and their parents. *AORN Journal*.

[2] Kain ZN, Mayes LC, Caldwell-Andrews AA, Karas DE, McClain BC (2006). Preoperative anxiety, postoperative pain, and behavioral recovery in young children undergoing surgery. *Pediatrics*.

[3] Litman RS (2011). Allaying anxiety in children: when a funny thing happens on the way to the operating room. *Anesthesiology*.

[4] Li HCW, Lopez V (2006). Assessing children’s emotional responses to surgery: a multidimensional approach. *Journal of Advanced Nursing*.

[5] Li HCW, Lopez V (2005). Children’s emotional manifestation scale: development and testing. *Journal of Clinical Nursing*.

[6] Craig KD, Versloot J, Goubert L, Vervoort T, Crombez G (2010). Perceiving pain in others: automatic and controlled mechanisms. *Journal of Pain*.

[7] Schiavenato M, Craig KD (2010). Pain assessment as a social transaction: beyond the “gold standard”. *Clinical Journal of Pain*.

[8] Breau LM, Burkitt C (2009). Assessing pain in children with intellectual disabilities. *Pain Research and Management*.

[9] Huguet A, Stinson JN, McGrath PJ (2010). Measurement of self-reported pain intensity in children and adolescents. *Journal of Psychosomatic Research*.

[10] Zhou H, Roberts P, Horgan L (2008). Association between self-report pain ratings of child and parent, child and nurse and parent and nurse dyads: meta-analysis. *Journal of Advanced Nursing*.

[11] Söderbäck M, Coyne I, Harder M (2011). The importance of including both a child perspective and the child's perspective within health care settings to provide truly child-centred care. *Journal of Child Health Care*.

[12] Hicks CL, Von Baeyer CL, Spafford PA, Van Korlaar I, Goodenough B (2001). The Faces Pain Scale—revised: toward a common metric in pediatric pain measurement. *Pain*.

[13] Stanford EA, Chambers CT, Craig KD (2006). The role of developmental factors in predicting young children’s use of a self-report scale for pain. *Pain*.

[14] von Baeyer CL, Uman LS, Chambers CT, Gouthro A (2011). Can we screen young children for their ability to provide accurate self-reports of pain?. *Pain*.

[15] McMurtry CM, Noel M, Chambers CT, McGrath PJ (2011). Children's fear during procedural pain: preliminary investigation of the Children's Fear Scales. *Health Psychology*.

[16] McGrath PJ, Walco GA, Turk DC (2008). Core outcome domains and measures for pediatric acute and chronic/recurrent pain clinical trials: PedIMMPACT recommendations. *Journal of Pain*.

[17] McGrath PA, Seifert CE, Speechley KN, Booth JC, Stitt L, Gibson MC (1996). A new analogue scale for assessing children’s pain: an initial validation study. *Pain*.

[18] Bringuier S, Dadure C, Raux O, Dubois A, Picot MC, Capdevila X (2009). The perioperative validity of the visual analog anxiety scale in children: a discriminant and useful instrument in routine clinical practice to optimize postoperative pain management. *Anesthesia and analgesia*.

[19] Fortier MA, Martin SR, MacLaren Chorney J, Mayes LC, Kain ZN (2011). Preoperative anxiety in adolescents undergoing surgery: a pilot study. *Paediatric Anaesthesia*.

[20] Kindler CH, Harms C, Amsler F, Ihde-Scholl T, Scheidegger D (2000). The visual analog scale allows effective measurement of preoperative anxiety and detection of patients’ anesthetic concerns. *Anesthesia and Analgesia*.

[21] Spielberger CD (1973). *Manual for the State-Trait Anxiety Inventory for Children*.

[22] Fortier MA, Chorney JM, Rony RYZ (2009). Children’s desire for perioperative information. *Anesthesia and Analgesia*.

[23] Apell J, Paradi R, Kokinsky E, Nilsson S (2011). Measurement of children's anxiety during examination or treatment in hospital—a study evaluating the short-STAI Vård i Norden. *Nursing Science & Research in the Nordic Countries*.

[24] Schisler T, Lander J, Fowler-Kerry S (1998). Assessing children’s state anxiety. *Journal of Pain and Symptom Management*.

[25] Marteau TM, Bekker H (1992). The development of a six-item short-form of the state scale of the Spielberger State-Trait Anxiety Inventory (STAI). *British Journal of Clinical Psychology*.

[26] Nilsson S, Kokinsky E, Nilsson U, Sidenvall B, Enskär K (2009). School-aged children’s experiences of postoperative music medicine on pain, distress, and anxiety. *Paediatric Anaesthesia*.

[27] Nilsson S, Hallquist C, Enskär K, Kokinsky E Active and passive distraction in children undergoing wound dressings.

[28] von Baeyer CL (2006). Children’s self-reports of pain intensity: scale selection, limitations and interpretation. *Pain Research and Management*.

[29] Miró J, Huguet A (2004). Evaluation of reliability, validity, and preference for a pediatric pain intensity scale: the Catalan version of the faces pain scale—revised. *Pain*.

[30] Murphy J, Cameron L (2006). *Talking Mats: A Resource to Enhance Communication*.

[31] Bornman J, Murphy J (2006). Using the ICF in goal setting: clinical application using Talking Mats. *Disability and Rehabilitation. Assistive Technology*.

[32] Murphy J (2006). Perceptions of communication between people with communication disability and general practice staff. *Health Expectations*.

[33] Murphy J, Tester S, Hubbard G, Downs M, MacDonald C (2005). Enabling frail older people with a communication difficulty to express their views: the use of Talking Mats as an interview tool. *Health and Social Care in the Community*.

[34] Cameron L, Murphy J (2002). Enabling young people with a learning disability to make choices at a time of transition. *British Journal of Learning Disabilities*.

[35] Ferm U, Sahlin A, Sundin L, Hartelius L (2010). Using Talking Mats to support communication in persons with Huntington’s disease. *International Journal of Language and Communication Disorders*.

[36] Widgit Software (1994). *Widgit Symbol*.

[37] Svensson E (2005). Choice and consequence: the measurement level determines the statistical toolbox. *Lakartidningen*.

[38] Stinson JN, Kavanagh T, Yamada J, Gill N, Stevens B (2006). Systematic review of the psychometric properties, interpretability and feasibility of self-report pain intensity measures for use in clinical trials in children and adolescents. *Pain*.

[39] Streiner D, Norman G (2008). *Health Measurement Scales—A practical Guide to Their Development and Use*.

[40] Wennström B (2011). *Experiences, Symptoms and Signs in 3-11 Year-Old Children Undergoing Day Surgery in the Context of Perioperative Dialogue*.

[41] Nguyen TN, Nilsson S, Hellström AL, Bengtson A (2010). Music therapy to reduce pain and anxiety in children with cancer undergoing lumbar puncture: a randomized clinical trial. *Journal of Pediatric Oncology Nursing*.

[42] Nilsson S, Björkman B, Almqvist AL, Almqvist L, Björk-Willén P, Donohue D Children's voices—differentiating a child perspective from a child's perspective.

[43] Caes L, Vervoort T, Eccleston C, Goubert L (2012). Parents who catastrophize about their child's pain prioritize attempts to control pain. *Pain*.

